# Test day variability in yield and composition of Surti and Mehsani buffaloes milk at day 15 and 60 postpartum

**DOI:** 10.14202/vetworld.2016.595-600

**Published:** 2016-06-16

**Authors:** K. K. Tyagi, B. P. Brahmkshtri, U. V. Ramani, V. B. Kharadi, G. M. Pandaya, M. Janmeda, K. J. Ankuya, M. D. Patel, L. M. Sorathiya

**Affiliations:** 1 Livestock Research Station, Navsari Agricultural University, Navsari, Gujarat, India; 2 Department of Animal Genetics and Breeding, College of Veterinary Sciences, Navsari Agricultural University, Navsari, Gujarat, India; 3 Department of Animal Biotechnology, College of Veterinary Sciences, Navsari Agricultural University, Navsari, Gujarat, India; 4 Livestock Research Station, Sardarkrushinagar Dantiwada Agricultural University, Sardarkrushinagar, Gujarat, India

**Keywords:** buffalo, Mehsani, milk composition, milk yield, Surti

## Abstract

**Aim:**

To estimate individual test day variability in yield and composition of Surti and Mehsani buffaloes milk at day 15 and 60 postpartum (pp).

**Materials and Methods:**

A total of 13 normally calved Surti and Mehsani buffaloes each maintained at Livestock Research Stations of Navsari and Sardarkrushinagar Dantiwada Agricultural Universities, respectively, were selected for the study. Milk sample was collected from each selected buffalo at day 15 and 60 pp to study milk yield and composition variability between these two breeds. Buffaloes were categorized for the ease of data analysis and comparisons into four groups, viz., S15 (Surti buffaloes 15^th^ day pp), S60 (Surti buffaloes 60^th^ day pp), M15 (Mehsani buffaloes 15^th^ day pp), and M60 (Mehsani buffaloes 60^th^ day pp).

**Results:**

There were 37.20% and 25.03% significant (p≤0.05) increase in mean test day milk yield (TDMY) of S60 and M60 as compared to S15 and M15 groups, respectively. The mean TDMY of Mehsani buffalo was 99.19% and 81.53% significantly (p≤0.05) higher than Surti buffaloes at day 15 and 60 pp, respectively. The mean fat and protein corrected test day milk yield (FPCTDMY) of all the groups was found to be significantly different (p≤0.05) from each other. There was significant (p≤0.05) increase of 1.94 and 3.45 kg in mean FPCTDMY with the progression of lactation between day 15 and 60 pp in Surti and Mehsani buffaloes, respectively. Similarly, the mean FPCTDMY of Mehsani buffaloes were approximately double with 103.27% and 96.36% higher yield as compared to Surti buffaloes at day 15 and 60 pp, respectively. Among milk composition, significant differences were observed for solid not fat (SNF) and protein%, whereas fat and lactose% were steady among four groups. The only significant (p≤0.05) difference was observed for SNF in M60 group, which was 8.29%, 6.85%, and 10.70% higher as compared to S15, S60, and M15 groups, respectively. The mean protein% in milk of Mehsani buffaloes was 21.01% and 33.05% significantly (p≤0.05) higher than Surti buffaloes milk at day 15 and 60 pp, respectively. However, there was a significant difference in protein% observed with the advancement of lactation in Mehsani buffaloes, but it was not so in the case of Surti buffaloes.

**Conclusion:**

Major consistent finding of the present study reveals that milk yield and protein% of Mehsani buffalo was significantly higher than Surti buffalo at day 15 and 60 pp.

## Introduction

Buffaloes are imperative sources of edible milk for human consumption in several parts of the world including India. The current buffalo population in India as per latest 19^th^ livestock census is 108.7 million which accounts for 21.23% of the total livestock population [1]. Gujarat had around 9.55% contemporary buffalo population of the country and bestowed with high milk producing breeds. Milk production in India grew at an annual growth rate of 5.0% and reached a volume of 127.9 million tons milk in the year 2011-2012 [2]. Buffalo was the prime contributor with 58.34% share to the total milk pool in Gujarat state [3]. Genetic improvement for milk production remained precedence of animal breeders for which they primarily utilize milk yield records.

One of the important milk yield records is test day milk yield (TDMY). Individual test date effect is one of the key advantages of using TDMY records. It becomes much more vital when gene expression studies interrelated to milk yield is sought by investigators on a particular day. Similarly, it is indispensable to know bioactive constituents of milk for maximum value addition. The composition of buffalo milk is mainly determined by fat, protein, solid not fat, (SNF) and lactose. Buffalo milk is characterized by higher solids contents for being a richer source of lipids, protein, lactose, and minerals.

Genetic variability remained the prime focus of breeders to explore reasons for differential production potential among different breeds. Keeping this in view, Surti and Mehsani buffalo breeds maintained at organized farms of state agricultural universities in their breeding tract were selected for the present study. The experiment was designed to understand TDMY and composition dynamics during early lactation in Surti and Mehsani buffaloes at organized farms situated in their home tracts.

## Materials and Methods

### Ethical approval

The prior approval from the Institutional Animal Ethics Committee was obtained for the use of Surti and Mehsani buffalo breeds maintained at Livestock Research Stations of Navsari and Sardarkrushinagar Dantiwada Agricultural Universities, respectively.

### Selection of experimental animals

About 13 normally calved Surti and Mehsani buffaloes each maintained at Livestock Research Stations of Navsari and Sardarkrushinagar Dantiwada Agricultural Universities, respectively, were selected for the study. Milk samples were collected repeatedly from the same buffaloes at 15^th^ and 60^th^ day postpartum (pp). Buffaloes were categorized for the ease of data analysis and interpretations into four groups, viz., S15 (Surti buffaloes 15^th^ day pp), S60 (Surti buffaloes 60^th^ day pp), M15 (Mehsani buffaloes 15^th^ day pp), and M60 (Mehsani buffaloes 60^th^ day pp).

### Sample collection

Whole milk sample from each selected animal was collected during milking into a sterile bucket, and milk yield was determined using electronic balance. 50 ml aliquot was taken in polypropylene tube and was subjected to milk composition analysis immediately after collection.

### Estimation of parameters

TDMY in kg was calculated by combining morning and evening milk yield of collection day. Cumulative milk yield in first 15 (CMY15) and 60 days pp (CMY60) was calculated by summing up TDMY of first 15 and 60 days pp, respectively. Milk composition of samples such as milk protein, fat, and SNF and lactose% was analyzed using Lactoscan milk analyzer as per manufacturer instructions. Fat and protein corrected TDMY (FPCTDMY) was calculated by correcting TDMY to 4.0% fat and 3.3% protein using the formula:

FPCTDMY (kg)= TDMY (kg)×[0.337+(0.116 ×fat%)+(0.06×protein%)] [4]

### Statistical analysis

The data on milk yield and milk composition was subjected to statistical analysis using Statistical Package for Social Sciences (SPSS, Version 20.0) software. Descriptive statistics specifying mean ± standard error of mean, highest and lowest value were calculated for each group. One-way ANOVA procedure was undertaken to compare means. *Post-hoc* multiple comparisons were made using Duncan multiple new range test. Independent sample t-test was used for two-group comparisons. Bivariate correlations were calculated using Pearson correlation coefficient. The size of correlation (very high, high, moderate, low, and negligible) was interpreted as per the standard classification [5].

## Results and Discussion

### TDMY

The TDMY recorded in present study ranged from 2.00 to 5.50 kg, 4.10 to 7.40 kg, 5.16 to 10.59 kg, and 6.49 to 12.52 kg among S15, S60, M15, and M60 groups, respectively. There were 37.20% and 25.03% significant (p≤0.05) increase in mean TDMY of S60 and M60 as compared to S15 and M15 groups, respectively. The mean TDMY of Mehsani buffalo was 99.19% and 81.53% significantly (p≤0.05) higher than Surti buffaloes at day 15 and 60 pp, respectively. This may be attributed to the superiority of genes accountable for milk production in Mehsani buffaloes compared to Surti buffaloes. In this context, the subsistence of the Mehsana breed in North Gujarat, India had been reported way back to 1940s. These buffaloes were obtained through selection of crossbred buffaloes having characteristics intermediate between Surti and Murrah [6,7]. The genetic preeminence of Murrah buffalo breed for milk yield in comparison to other buffalo breeds had been advocated by several authors [8-10]. Thus, higher milk yield in Mehsani buffaloes in comparison to Surti buffaloes may partially be credited to introgression of higher milk-producing genes from Murrah buffaloes into this breed and their ensuing fixation in gene pool. Steady significant increase in milk yield was observed from 15^th^ day (2^nd^ week) to 60^th^ day (9^th^ week) in Surti and Mehsani buffaloes with the progression of lactation. The similar steady increasing trend of TDMY with 5.52±0.06 kg in 2^nd^ week to 7.90±0.06 kg in 9^th^ week of lactation had been reported in Murrah buffaloes [9]. Milk production was also found to augment between days 16 and 60 (11.35 kg/head) in Mediterranean buffaloes [11]. Similarly, a raise of 6.63 kg in weekly milk yield from 2^nd^ week (29.64 kg) to 9^th^ week (36.27 kg) had also been reported in Nagpuri buffaloes [12].

### Fat and protein corrected test day milk yield (FPCTDMY)

The mean FPCTDMY of all the groups was found to be significantly different (p≤0.05) from each other. The range of FPCTDMY observed among S15, S60, M15, and M60 groups was 2.84-7.60, 5.66-9.64, 7.41-15.21, and 10.06-18.47 kg, respectively. There was significant (p≤0.05) increase of 1.94 and 3.45 kg in mean FPCTDMY with the advancement of lactation between day 15 and 60 pp in Surti and Mehsani buffaloes, respectively. Correspondingly, the mean FPCTDMY of Mehsani buffaloes were almost double with 103.27% and 96.36% higher yield as compared to Surti buffaloes at day 15 and 60 pp, respectively. The mean FPCTDMY reflected genuine milk yield irrespective of protein and fat contents of the milk because these two major factors were corrected using a formula [4]. The mean FPCTDMY was 40.16%, 40.28%, 43.03%, and 51.73% higher as compared to mean TDMY of S15, S60, M15, and M60 groups, respectively. This can be due to higher protein and fat% in Surti and Mehsani buffaloes milk compared to normalized values used in correction formula. The percent increase for mean FPCTDMY compared to mean TDMY was higher in Mehsani buffaloes because of their plausibly higher protein and fat% increase with progression of lactation (Table-1).

**Table-1:** Mean milk yield and composition traits of Surti and Mehsani buffaloes at day 15 and 60 postpartum.

Traits/Groups	S15	S60	M15	M60	F/t values
				
N	13	13	13	13
TDMY (kg)	3.71^a^±0.26	5.09^b^±0.26	7.39^c^±0.45	9.24^d^±0.48	42.43**
FPCTDMY (kg)	5.20^a^±0.37	7.14^b^±0.35	10.57^c^±0.62	14.02^d^±0.71	52.69**
CMY15 (kg)	52.04^a^±4.00	-	97.27^b^±5.33	-	6.79**
CMY60 (kg)	-	255.55^a^±14.60	-	477.52^b^±24.46	7.79**
Fat%	7.34±0.11	7.39±0.20	7.2±0.14	7.79±0.16	2.63
SNF%	9.65^a^±0.09	9.78^a^±0.10	9.44^a^±0.14	10.45^b^±0.17	11.48**
Protein%	3.57^a^±0.04	3.51^a^±0.04	4.32^b^±0.05	4.67^c^±0.07	124.14**
Lactose%	5.12±0.07	5.24±0.09	5.07±0.06	5.32±0.08	2.20

** highly significant at p≤0.01,

N=Number of observations. Means bearing different superscripts between groups differed significantly. TDMY=Test day milk yield, FPCTDMY=Fat and protein corrected test day milk yield, CMY15=Cumulative milk yield in first 15 days pp, CMY60=Cumulative milk yield in first 60 days pp, SNF=Solid not fat

### Cumulative milk yield of first 15 and 60 days

The CMY15 had ranged from 28.26-87.14 to 73.25-134.15 kg among Surti and Mehsani buffaloes, respectively. The lowest to highest CMY60 observed among Surti and Mehsani buffaloes were 172.06-384.44 kg and 364.58-641.45 kg, respectively. The mean CMY15 and CMY60 of Mehsani buffalo were just about 87% significantly (p≤0.05) higher than Surti buffalo. Higher, mean CMY60 (313.89 kg) had been reported in Surti buffaloes maintained at Livestock Research Station, Vallabhnagar, Rajasthan [13]. This may be ascribed to variation in various non-genetic factors such as location, period of study, size of data sets, and parity. In the present study, the CMY15 and CMY60 were also significantly higher in Mehsani as compared to Surti buffaloes. These findings were in agreement with the higher standard lactation yield of 1984.17±81.78 kg reported for Mehsani buffaloes [14] as compared to 1450.07±43.99 kg of Surti buffaloes [15]. Lactation yield of 1610 kg (1308-1838) was also reported for Mehsani buffaloes under Indian conditions [16]. Average milk production of around 2000 kg per lactation with lactation length of 315 days in Mehsani buffaloes had also been cited [17]. The higher cumulative milk yield in Mehsani buffaloes compared to Surti buffaloes may be endorsed to various genetic and non-genetic factors as discussed previously for similar differences observed in TDMY and FPCTDMY in these two breeds.

### Milk composition

The milk composition in Surti and Mehsani buffaloes at different stages of lactation was analyzed. While reviewing, it was observed that data pertaining to milk yield at different lactation stages were available; however, very few studies had described composition traits around day 15 and 60 pp. Accordingly, data obtained in the present study have been discussed about the overall and stage wise composition data available in different lactation studies.

### Fat%

The fat% was found to be steady without any notable significant differences among the four groups. The fat% ranged from 6.23% to 7.92%, 6.52% to 8.82%, 6.46% to 7.99%, and 6.92% to 8.80% in milk of S15, S60, M15, and M60 groups, respectively. In the present study, the mean fat% among S15, S60, M15, and M60 groups was in the range of 7.2-7.79%. The mean fat% among the groups did not differ significantly. The mean fat% data observed under this study was higher than earlier published reports of 6.17% and 6.46% for Surti and Mehsani buffaloes [18]. However, fat% reported in the present study was lower than 8.59%, 8.10%, and 8.40% fat reported for water buffaloes in Italy [19], Turkey [20], and Europe [21], respectively. Some of the reports of lower fat (6.59%) as compared to present study were also reported in Mediterranean breed [22]. Complementary to our results, significantly higher fat content (8.64%) at the end of the peak production period in Mediterranean buffaloes was also been reported [11]. Compared to four groups of present study, the higher fat contents of 8.71% [23] and 9.01% [24] were also reported in Mediterranean buffaloes. Nevertheless, comparable fat (7.0-7.7%) was reported in other several studies [25-29].

### SNF%

The lowest SNF% observed was 9.30%, 9.25%, 6.46%, and 8.83% among milk of S15, S60, M15, and M60 groups, respectively. The highest SNF% observed was 10.45%, 10.38%, 10.15%, and 11.27% among milk of S15, S60, M15, and M60 groups, respectively. The only significant (p≤0.05) difference was observed in the M60 group which was 8.29, 6.85, and 10.70% higher as compared to S15, S60, and M15 groups, respectively. Mean SNF content obtained in milk of S15, S60, and M15 groups in our experiment was nearly similar to mean SNF of 9.57±0.03% during the first month reported in Murrah buffaloes [30]. However, in contrast to the present study, they reported a decrease in mean SNF to 9.38±0.03% in successive second month. The mean SNF of 9.4-9.5% roughly similar to that of M15 group had been reported in Murrah buffaloes of Punjab [31] and Swamp buffaloes of Bangladesh [32]. Although, mean SNF of 9.9% that comes in between the mean SNF% reported in the present study for S60 and M60 was also reported in Bulgarian buffaloes [33]. The higher mean SNF of 10.45% had been observed for the M60 group in the present study. Earlier a range of 9.8-10.1% SNF had been reported for Egyptian buffaloes [34], which was nearly comparable to the SNF% reported in the present study among S60 and M60 group. Mean SNF of 8.3% compared to all four groups of present study had also been reported in non-descript buffaloes reared at high altitudes in the Kumaon hills of the central Himalayas [35]. Similarly, mean SNF of 9.2% had also been reported in water buffaloes of Bangladesh [32]. The only reference of high SNF% comparable to that of M60 group was cited in Brazilian buffaloes producing milk with 10.4% SNF [22].

### Protein%

The mean protein% in milk of Mehsani buffaloes was 21.01% and 33.05% significantly (p≤0.05) higher than Surti buffaloes milk at day 15 and 60 pp, respectively. However, there was a significant difference in protein% observed with the advancement of lactation in Mehsani buffaloes, but it was not so in the case of Surti buffaloes. There was 8.10% significant (p≤0.05) increase in mean protein% observed from day 15 to 60 pp in Mehsani buffalo. The protein% ranged from 3.30% to 3.92%, 3.36% to 3.71%, 4.05% to 4.62%, and 4.33% to 5.06% among milk of S15, S60, M15, and M60 groups, respectively. The lowest mean protein content in the present study was observed in the S60 group and that of highest was observed in M60 group. The protein% observed in the present study was higher than 2.70% protein reported in non-descript buffaloes reared at high altitudes in the Kumaon hills of the central Himalayas [35]. Slightly higher protein (3.6-3.85%) as compared to S15 and S60 were reported in Egyptian buffaloes [34,36]. Protein content within the range of 3.73-3.97% which was in between the observed protein% in milk of Surti and Mehsani buffaloes had been reported in Murrah buffaloes of India [30,31] and Argentina [37,38] as well as water buffaloes of Bangladesh [32]. Slightly lower protein of 4.11% and 4.13% as compared to the M15 group was reported in buffaloes of Pakistan [39] and Brazil [22], respectively. The protein content of 4.24-4.45% in Azerbiajan [40], while 4.35% in buffaloes of Pakistan [41] and France [27], comparable to M15 had also been reported. The protein content of 4.40% and 4.49% in between the range of M15 and M60 group was reported in buffaloes of Turkey [42] and Bulgaria [33], respectively. The protein content of 4.74% and 4.65% comparable to that of M60 group was reported in buffaloes of China [43] and Italy [44]. Higher protein% as compared to the present study to the tune of 4.90% and 5.20% had also been reported in buffaloes of Germany [45] and Jaffarabadi buffaloes reared in Argentina [38], respectively.

### Lactose%

The lowest to highest lactose% observed was 4.69-5.54%, 4.85-5.80%, 4.84-5.51%, and 5.02-5.86% among milk of S15, S60, M15, and M60 groups, respectively. There was no significant difference observed for mean lactose% within and between breeds. Lactose% in the range of 4.99-5.24 was reported in buffaloes of Egypt [34,36] that are in agreement with the findings of the present experiment. Lactose% in between 4.5% and 5.0% had been reported in buffaloes of India [30,31], Italy [44], Pakistan [39], Bangladesh [32], Azerbiajan [40], Murrah breed in Argentina [37,38], Jaffarabadi breed in Argentina [38], and Bulgaria [33]. However, lactose% higher than 5.0% in concurrence to present study was also reported in buffaloes of Pakistan [41] and France [27].

### Correlation coefficients among milk yield and composition traits

The correlation coefficients among milk yield and composition traits of Surti and Mehsani buffaloes are presented in Tables-2 and 3. The moderate positive significant (p≤0.05) correlation was observed between protein and fat% in Surti buffaloes at day 60 pp. However, at day 60 pp moderate positive non-significant correlation was observed between protein and SNF% in Mehsani buffaloes. The only moderate positive non-significant correlation at day 15 was observed in Surti buffaloes between TDMY and lactose%. All remaining correlations were of low to the negligible magnitude between different traits among the four groups.

**Table-2:** Correlation coefficients between milk yield and composition traits at day 15 (above diagonal) and day 60 postpartum (below diagonal) in Surti buffaloes.

Traits	TDMY (kg)	%			

		Fat	SNF	Protein	Lactose
TDMY (kg)	-	0.02	−0.16	0.25	0.52
Fat%	−0.27	-	0.26	−0.22	0.19
SNF%	−0.15	0.26	-	−0.09	0.17
Protein%	0.33	0.63*	0.22	-	−0.30
Lactose%	0.01	−0.22	0.24	0.14	-

* Significant at p≤0.05.

TDMY=Test day milk yield, SNF=Solid not fat

**Table-3:** Correlation coefficients between milk yield and composition traits at day 15 (above diagonal) and day 60 postpartum (below diagonal) in Mehsani buffaloes.

Traits	TDMY (kg)	%			

		Fat	SNF	Protein	Lactose
TDMY (kg)	-	−0.33	−0.35	0.36	0.15
Fat%	−0.36	-	0.33	0.05	0.08
SNF%	−0.22	0.46	-	−0.11	−0.18
Protein%	0.28	0.36	0.53	-	−0.09
Lactose%	0.01	−0.06	0.13	0.21	-

TDMY=Test day milk yield, SNF=Solid not fat

Similar to S60 group, a significant correlation between fat and protein% had also been reported with moderate positive significant correlation (0.60) in mixed buffalo milk [46]. Most of the correlations in the present study were low to the negligible magnitude and were non-significant to draw out any valid and repeatable inference. Comparable non-significant correlations of low to negligible magnitude among milk yield and composition traits had also been reported by several authors [46-48].

## Conclusion

In the present study, it was found that Mehsani buffaloes were significantly higher milk producer in terms of TDMY and FPCTDMY as compared to Surti buffaloes at day 15 and 60 pp. The major consistent finding of the present study reveals that milk yield and protein% of Mehsani buffalo was significantly higher than Surti buffalo at day 15 and 60 pp.

## Authors’ Contributions

KKT, BPB, UVR, and VBK designed the study. The experiment was done by KKT, GMP, MJ, KJA, MDP, and LMS, whereas laboratory work was done by KKT, GMP, and MJ. All the authors participated in data analysis, draft, and revision of the manuscript. All authors read and approved the final manuscript.
